# Mycobacterium sp. as a possible cause of hypersensitivity pneumonitis in machine workers.

**DOI:** 10.3201/eid0502.990213

**Published:** 1999

**Authors:** B G Shelton, W D Flanders, G K Morris

**Affiliations:** PathCon Laboratories, Norcross, Georgia 30092, USA. bshelton@pathcon.com

## Abstract

Hypersensitivity pneumonitis (HP) in workers exposed to metal removal fluids (MRFs) is increasing. This study supports the hypothesis that aerosolized mycobacteria colonizing the MRFs likely cause the disease. Three case studies of HP outbreaks among metal workers showed potentially high exposures to a rare and newly proposed Mycobacterium species. Retrospective review of samples submitted to our laboratory showed an association between presence of mycobacteria and HP.


### *Mycobacterium* sp. as a Possible Cause of Hypersensitivity Pneumonitis in Machine Workers

**Brian G. Shelton,* W. Dana Flanders,† and George K. Morris***
*PathCon Laboratories, Norcross, Georgia, USA; and
†Emory University, Atlanta, Georgia, USA

Hypersensitivity pneumonitis (HP) in workers exposed to metal removal fluids (MRFs) is increasing. This study supports the hypothesis that aerosolized mycobacteria colonizing the MRFs likely cause the disease. Three case studies of HP outbreaks among metal workers showed potentially high exposures to a rare and newly proposed *Mycobacterium* species. Retrospective review of samples submitted to our laboratory showed an association between presence of mycobacteria and HP.

Interest in occupational hypersensitivity pneumonitis (HP) or extrinsic allergic alveolitis is increasing (1,2). HP, a rare group of diseases characterized by recurrent dyspnea, cough, and systemic signs such as myalgia and fever, is caused by repeated exposure and subsequent sensitization to various antigens. Recognized antigens are often fungal or bacterial. The disease process is thought to involve lymphocyte-sensitization and cell-mediated immune response that ultimately results in alveolitis. Types of occupational HP include farmer's lung, bird fancier's lung, and mushroom worker's lung.

HP has recently been recognized among metal workers, and as evidenced at a recent meeting of the Automotive Manufacturers Association in Detroit (September 1997), controversy exists as to its possible cause(s). Researchers from the National Institute for Occupational Safety and Health recently reported that four of six outbreaks of HP in metal-working facilities yielded unusual flora (e.g., *Mycobacterium chelonae*) in contaminated MRFs (2,3). They discussed acid-fast bacteria, nontuberculous mycobacteria, gram-positive bacteria, and fungi as possible causes (3) but more recently concluded that the specific etiologic agent(s) for HP among workers exposed to (MRF) aerosol remain(s) unknown (2). Advances in metal removal fluid technology have led to the use of synthetic, semisynthetic, and soluble fluids, as opposed to traditional oil coolants. These water-based coolants are typically recycled and often become colonized by microorganisms. We present case reports and observations that further suggest mycobacteria may be a cause of occupational HP.

Address for correspondence: Brian G. Shelton, PathCon Laboratories, 270 Scientific Drive, Suite 3, Norcross, Georgia 30092, USA: fax: 770-446-0610; e-mail: bshelton@pathcon.com.

### Case Report 1

We investigated a single case of HP in an employee who worked in an engine production facility in 1997. Other than contaminated fluids, no chemicals or substances known to cause HP were used at the facility. This 45-year-old male nonsmoker, in otherwise good health, worked in a wet machining area of the shop. His symptoms, which manifested themselves while he was at work, included cough, dyspnea, and hoarseness. One episode, which included malaise and fever of 102°F, led to hospitalization. In-hospital evaluation included a chest X-ray, which showed bilateral interstitial infiltrates, a high resolution computed tomography scan consistent with alveolitis, normal spirometry with a decreased diffusing capacity for carbon monoxide, and nonspecific transbronchial biopsy findings. A diagnosis of HP was made, and the patient was treated with oral glucocorticoids and relocated to an office environment at work. His symptoms gradually improved over several weeks, and the steroids were discontinued.

Mycobacteria in the *M. chelonae* complex were identified in high numbers in numerous bulk coolant samples (from nondetectable to 6.6x$10^6$ CFU/ml) and counts from 56 to 2,256 CFU/$m^3$ in positive air samples around the colonized machines. Other organisms identified in the bulk samples included *Pseudomonas pseudoalcaligenes*, *P. alcaligenes*, and yeast with counts ranging from nondetectable to 1.7x$10^6$ CFU/ml. The *Mycobacterium* isolates were identical to *M. immunogen* (4), a proposed rare and new species of rapidly growing mycobacteria with similarities to both *M. chelonae* and *M. abscessus*. The case-patient (Case 1) and an unaffected co-worker (Control 1) yielded a significantly elevated antibody immunoglobulin G (IgG) titer against this *Mycobacterium* and other organisms isolated from coolant samples at the facility. Five other unaffected workers (Controls 2-6) who worked in the same area as the case-patient were tested, as were 23 other controls comprising the reference population not known to have exposure to the facility. The standard deviation index used in the Figure was calculated by subtracting the mean IgG/ml level of the reference population from the patient's IgG/ml. This value was then divided by the standard deviation of the reference population to give the standard deviation index. For example, the levels were 5 or more in the case-patient, indicating that his levels were at least 5 standard deviations higher than the corresponding mean antibody levels in the reference population. The serum antigen-specific antibody levels were measured by enzyme-linked immunosorbent assay.

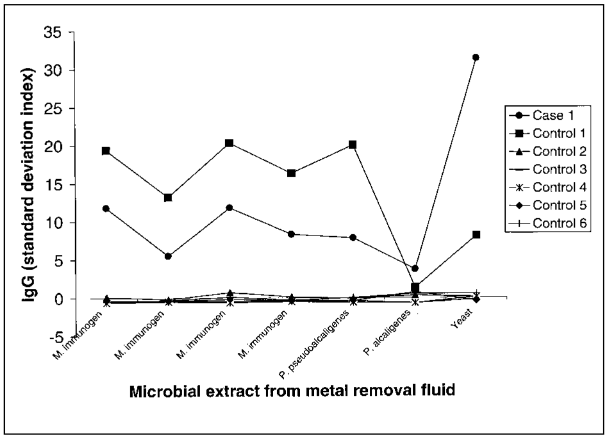

Figure. Immunoglobulin G (IgG) antibody titer vs. microbial extract from metal removal fluid.

### Case Report 2

We were also involved in the investigation of HP at an engine manufacturing plant that had numerous machines for cutting and grinding metal. The facility employed approximately 700 machine workers. After HP was diagnosed by a treating pulmonologist in two middle-aged male machinists, the facility was inspected for known causes of HP. No causal exposures were identified other than exposure to cutting fluids, which was common to the two cases. Samples of machine fluid samples yielded *Mycobacterium* colony counts of $10^2$ to $10^7$ per ml, with mycobacteria being the most predominant organism in some fluids. Air samples near colonized machines yielded high counts of mycobacteria, some of which exceeded the upper limits of the sampler (>9,424 CFU/$m^3$ of air). Other organisms in air samples included *Corynebacterium*, *Klebsiella*-like organisms, *Bacillus*, *Rhodococcus*, *Hyalodendron*, *Cladosporium*, and nonsporulating fungi at levels of 24 CFU/$m^3$ to 1,319 CFU/$m^3$. No mycobacteria were detected immediately outside the facility. The mycobacteria detected were rapid growers identical to the newly proposed species *M. immunogen*. We evaluated a sister plant (in the same state) that had no known cases of HP; no mycobacteria were detected from samples of cutting fluids.

### Case Report 3

We are currently investigating a cluster of five cases of physician-diagnosed HP at a large metal working factory in the midwestern part of the United States. Other than contaminated fluids, no chemicals or other substances known to cause HP are used at the facility. Samples of MRFs showed counts of mycobacteria ranging from nondetectable to >$10^6$ CFU/ml of sample. Again, the *Mycobacterium* species was identical to the newly proposed species, *M. immunogen*. Other organisms identified in bulk samples included *Bacillus*, *Pseudomonas*, *Cladosporium*, *Trichoderma*, and nonsporulating fungi at concentrations of <10 CFU/ml to 6,000 CFU/ml.

### Conclusions

All the cases we report occurred in men exposed to metal removal fluids heavily colonized with mycobacteria. Moreover, we have documented high airborne concentrations of mycobacteria in the workplace. At least five other clusters of HP in workers exposed to mycobacteria have been reported (2,3,5-7). In one outbreak of HP in metal workers (6,7), a sputum culture sample from one of the six patients in the outbreak yielded *M. chelonae*—a possibly rare event in a person without pulmonary infection attributed to that organism. By documenting further clusters of HP among workers exposed to MRF contaminated with mycobacteria, our findings provide an additional link suggesting a possible association between mycobacteria and HP.

It is possible that mycobacteria commonly contaminate MRFs even in the absence of HP. To assess this possibility, we identified all samples of MRF that had been submitted for culture to our laboratory since 1993 when we routinely started testing these samples for mycobacteria. Of the seven facilities from which we received samples as part of an investigation of known HP, six had mycobacteria. Of the eight facilities without known HP, only one had mycobacteria (odds ratio = 42, 95% confidence interval = 1.5-2194.47, p = 0.01 by Fisher's exact test). Thus, in facilities without HP, mycobacteria were not commonly found in MRFs. Even though they represent a review of existing records and not a prospectively designed study, these data suggest that mycobacterial contamination of MRFs may increase the risk for HP.

The hypothesis that mycobacterial contamination in cutting fluids is a cause of HP has high biologic plausibility supported by several observations: Cutting fluids and their contaminants can become easily aerosolized thus providing a high opportunity for inhalation exposure; mycobacteria have an acid-fast cell wall that is highly antigenic; mycobacterium adjuvants, often used by immunologists to elicit a stronger immunologic reaction, may lead to granuloma formation if used more than once; elevated IgG antibody levels directed at mycobacteria were found in patients with HP—the case-patient in case report 1 had elevated antibody titers to several antigens including mycobacteria in the MRFs (although not diagnostic of HP, this elevated titer documents exposure and immunologic response to mycobacteria in this patient); outbreaks of HP in hot tubs have been reported with mycobacteria (*M. avium*) as the suspect cause (8); and animal models used to study HP have been produced by using antigenic extracts from mycobacteria (9).

This hypothesis—mycobacteria as an antigen, or as an adjuvant that enhances reactions to other antigens found in the environment—is biologically plausible. Mycobacteria may be an important cause of HP in occupational settings or other settings in which exposure to contaminated aerosolized aqueous solutions is possible. These data and observations alone neither prove nor disprove a causal link. However, given the available data from the case studies presented here and elsewhere (2,3,5-7), the ability of *Mycobacterium* to proliferate to high levels in certain water-based MRFs, biologic plausibility, and the lack of use of other substances known to cause HP at these facilities, exposure to mycobacteria should be considered as a possible cause.

Dr. Shelton is vice president of PathCon Laboratories, a private laboratory specializing in the investigation of airborne environmental microorganisms. His research interests are in the epidemiology of environmental microorganisms in building water systems and indoor air. He has conducted numerous outbreak investigations and worked on the prevention and control of Legionnaires' disease. He was the first to use environmental concentrations of Legionella to predict disease risk.

### References

1. Centers for Disease Control and Prevention. Biopsy-confirmed hypersensitivity pneumonitis in automobile production workers exposed to metalworking fluids—Michigan, 1994-1995. MMWR Morb Mortal Wkly Rep 1996;45:606-10.
2. Centers for Disease Control and Prevention. Criteria for a recommended standard: occupational exposure to metalworking fluids. Cincinnati (OH): NIOSH; 1998. Publication No. 98-102.
3. Kreiss K, Cox-Ganser J. Metalworking fluid-associated hypersensitivity pneumonitis: a workshop summary. Am J Ind Med 1997;32:423-32.
4. Wilson RW, Steingrube VA, Bottger EC, SpringerB, Brown BA, Jost KC, et al. Recognition of a new taxon within the *Mycobacterium abscessus-Mycobacterium chelonae* complex and proposal of *Mycobacterium immunogen* sp. nov. [abstract #16]. Washington: American Society for Microbiology; 1998.
5. Zacharisen MC, Kadambi AR, Schlueter DP, Kurup VP, Shack JB, Fox JL, et al. The spectrum of respiratory disease associated with exposure to metal working fluids. J Occup Environ Med 1998;40:640-7.
6. Bernstein DI, Lummus ZL, Santilli G, Siskosky J, Bernstein IL. Machine operator's lung. A hypersensitivity pneumonitis disorder associated with exposure to metalworking fluid aerosols. Chest 1995;108:636-41.

7. Muilenburg ML, Burge HA, Sweet T. Hypersensitivity pneumonitis and exposure to acid-fast bacilli in coolant aerosols [abstract #683]. J Allergy Clin Immunol 1993;91:311.
8. Embil J, Warren P, Yakrus M, Stark R, Corne S, Forrest D, et al. Pulmonary illness associated with exposure to *Mycobacterium-avium* complex in hot tub water. Hypersensitivity pneumonitis or infection? Chest 1997;111:813-6.
9. Richerson HB, Suelzer MT, Swanson PA, Butler JE, Kopp WC, Rose EF. Chronic hypersensitivity pneumonitis produced in the rabbit by the adjuvant effect of inhaled muramyl dipeptide (MDP). Am J Pathol 1982;106:409-20.

## References

[PDF_00275] Bernstein D. I., Lummus Z. L., Santilli G., Siskosky J., Bernstein I. L. (1995). Machine operator's lung. A hypersensitivity pneumonitis disorder associated with exposure to metalworking fluid aerosols.. Chest.

[PDF_00253] Centers for Disease Control and Prevention (CDC) (1996). Biopsy-confirmed hypersensitivity pneumonitis in automobile production workers exposed to metalworking fluids--Michigan, 1994-1995.. MMWR Morb Mortal Wkly Rep.

[PDF_00286] Embil J., Warren P., Yakrus M., Stark R., Corne S., Forrest D., Hershfield E. (1997). Pulmonary illness associated with exposure to Mycobacterium-avium complex in hot tub water. Hypersensitivity pneumonitis or infection?. Chest.

[PDF_00262] Kreiss K., Cox-Ganser J. (1997). Metalworking fluid-associated hypersensitivity pneumonitis: a workshop summary.. Am J Ind Med.

[PDF_00271] Zacharisen M. C., Kadambi A. R., Schlueter D. P., Kurup V. P., Shack J. B., Fox J. L., Anderson H. A., Fink J. N. (1998). The spectrum of respiratory disease associated with exposure to metal working fluids.. J Occup Environ Med.

